# The Role of Diminishing Appetite and Serum Nesfatin-1 Level in Patients with Burn Wound Infection

**DOI:** 10.5812/ircmj.4198

**Published:** 2013-05-05

**Authors:** Ayse Albayrak, Ismail Demiryilmaz, Yavuz Albayrak, Belkiz Aylu, Bunyami Ozogul, Serkan Cerrah, Muhammed Celik

**Affiliations:** 1 Department of Infectious Diseases and Clinical Microbiology, Erzurum Region Education and Research Hospital, Erzurum, Turkey; 2 Department of General Surgery, Ibn-i Sina Hospital, Turkey; 3 Department of General Surgery and Burn Unit, Erzurum Region Education and Research Hospital, Erzurum, Turkey; 4 Department of Internal Medicine, Ataturk University, Faculty of Medicine, Erzurum, Turkey; 5 Department of Biochemistry, Ataturk University, Faculty of Medicine, Erzurum, Turkey

**Keywords:** Burns, Wound Infection, Nucleobindin, Appetite

## Abstract

**Background:**

The burn wound represents a susceptible site for opportunistic colonization by organisms of endogenous and exogenous origin. Diminishing appetite is known to occur in patients with burn infection, yet its underlying reason is not fully understood. We have examined the levels of nesfatin 1, a protein that we consider to be a potential new treatment target for the solution of appetite and nutrition problem in patients with burn infection.

**Objectives:**

The aim of the present study was therefore to examine nesfatin levels in patients with burn infection.

**Material and Methods:**

Laboratory values, medication and dietary records, and patient notes with diagnostic information of burn wounds patients who were admitted to the Division of Burn Treatment Center were obtained from the Erzurum Region Education and Research Hospital electronic database. Post-burn wound infection was objectively assessed by culturing wound homogenates from skin tissue. The main immediate inflammatory stress response parameters assessed were serum CRP concentrations, WBC counts, and blood nesfatin concentrations.

**Results:**

Scalding was the predominant cause of burns in both categories of patients. In 19 (61.3%) burn wound infection patients, the burns were due to a scald. A significant difference was found for the nesfatin, CRP, and WBC levels between the patients and the control group (P = 0.000). A significant difference was also determined between the nesfatin, CRP, and WBC figures at the time of hospitalization and at discharge from the hospital (P = 0.000). The most predominant bacterial isolate was Pseudomonas aeruginosa 16 (51.6%) followed by Methicilline resistant *Staphylococcus aureus* (MRSA) 7 (22.6%).

**Conclusions:**

We showed that the serum nesfatin 1 level was significantly lower in the patients with burn than in the control group in our study. We considered that the central nesfatin 1 system should be taken into consideration, rather than the peripheric nesfatin 1 system, when considering the regulation of appetite in patients with burns and particularly those accompanied by infection. In other explanation of the observed negative correlation between nesfatin 1 and burn wound infection suggests that nesfatin 1 may indicate the possible contribution of nesfatin 1 to the energy homeostasis.

## 1. Background

Burn injury is a serious trauma and can cause profound metabolic changes, including significant increases in catecholamines and corticosteroids, and a generalized catabolic response in the human body (1). The dead and denatured burn eschar and the moist wound environment favor colonization and proliferation of a variety of microorganisms (2). Burn injury causes depression of the immune response and severe catabolism proportional to the extent of injury (3, 4). The central nervous system (CNS) contains a number of neuroactive molecules that regulate energy homeostasis. Appetite-regulating molecules in the CNS are categorized as either anorexigenic or orexigenic, depending on their function. Nesfatin-1, a novel 82-amino acid peptide derived from nucleobindin-2, has recently been identified as a satiety peptide (5, 6). Nesfatin-1 is widely distributed in the CNS and in peripheral tissues such as adipose tissue, pancreas, and stomach (5-7). It is involved in a wide range of physiological functions including food intake, appetite stimulation, and energy homeostasis, and its expression is decreased in the hypothalamic paraventricular nucleus during starvation (6, 8).

## 2. Objectives

Diminishing appetite is known to occur in patients with burn infection, yet its underlying reason is not fully understood (1). Inflammatory processes increase in patients with burn infection and could suppress appetite increasing pathways. The aim of the present study was, therefore, to examine nesfatin levels in patients with burn infection.

## 3. Patients and Methods

A total of thirty one burn wounds patients who were admitted to the Division of Burn Treatment Center (level II burn treatment center), Erzurum Region Education and Research Hospital, from June 2010 to June 2011, were included in this study. Patients with a pre-existing or new diagnosis of metabolic diseases (such as diabetes mellitus, Cushing syndrome, acromegaly, etc.), liver disease, renal failure, or any infectious diseases (such as urinary tract, respiratory system, or soft tissue infection) known to independently influence C-reactive protein (CRP), white blood cell (WBC), or nesfatin production were excluded from the study. Patients who received insulin, glucocorticoids, or total parenteral nutrition during their hospitalization were also excluded because of the known influence of these interventions on CRP, WBC, and nesfatin levels. Those whose total burned surface area (TBSA) are higher than 10% were taken into the study. Laboratory values, medication and dietary records, and patient notes with diagnostic information were obtained from the Erzurum Region Education and Research Hospital electronic database. Post-burn wound infection surveillance was made in the burn tissue by the regular monitoring of the tissue biopsy samples and surface cultures. The burn tissue was followed according to the definitive guide of burn infections that are defined by the National Nosocomial Infections Surveillance System (NNIS). Gram-staining was performed of representative colonies to identify the type of infectious bacteria. The main immediate inflammatory stress response (injury response) parameters assessed were serum CRP concentrations, WBC counts (inflammatory response), and blood nesfatin concentrations (metabolic response). The whole blood from healthy controls or from patients within the first twelve hours after trauma (day 0) was collected into sterile tubes at day 0 (upon presentation at the hospital) and was taken after diagnosing burn injury infection (3-21st days). Serum nesfatin levels were measured using the residual blood from the sera taken for routine examination. All samples were tested with an immunoenzymatic method in a blinded fashion. Serum nesfatin concentrations were determined using enzyme immunoassay kit (EIA) designed to detect Nesfatin peptide based on the principle of Competitive Enzyme Immunoassay (The RayBio®, RayBiotech, Inc. Norcross, Georgia, USA). CRP, and WBC were measured by the standard assays. Statistical analysis was performed using the SPSS software version 19.0. The variables were investigated using visual (histograms, probability plots) and analytical (the Shapiro-Wilk test) methods to determine normal distribution. Descriptive analyses were presented using means and standard deviations (mean ± SD) for normally distributed variables (CRP and WBC). A students’s paired t-test was used to compare the measurements of CRP and WBC at two time points (upon presentation at the hospital and when treatment was terminated). Nesfatin values were presented using medians and interquartile range (IQR) for the non-normally distributed and ordinal variables. The Wilcoxon test was used to compare the changes in nesfatin occurring between the time of presentation at the hospital and when the treatment was terminated. In addition, since the nesfatin measurements were not normally distributed, nonparametric tests were conducted to compare these parameters as well as to compare the ordinal variables. The Mann-Whitney U test was used to compare nesfatin levels between the patients and the controls. The correlation coefficients and their significance were calculated using the Spearman test. A p-value of less than 0.05 was considered statistically significant.

## 4. Results

Study enrollment included thirty one patients who met the entrance criteria for this study [fifteen males (48.4%) and sixteen females (51.6%)]. Patient age ranged from 1 to 66 years, with an average age of 15.0 ± 18.6 years. The clinical characteristics of the patient population studied are shown in (Table 1). Scalding was the predominant cause of burns in both categories of patients. In nineteen (61.3%) burn wound infection patients, the burns were due to a scald, in five (16.1%), they were due to an electrical injury, in four (12.1%) they were due to a flame, and in three (9.7%) they were due to a tandir (oven) burn. The TBSA among the patients varied from 11 to 42%, with an average of 23.4 ± 9.2 (%). Nesfatin, CRP, and WBC levels measured during the hospitalization of the patients with burn infection and the values of the control group are shown in (Table 2). A significant difference was found for the WBC, CRP, and nesfatin levels between the patients and the control group (P = 0.000). A significant difference was also determined between the WBC, CRP, and nesfatin levels at the time of hospitalization and at discharge from the hospital (P = 0.000) (Table 3). No correlation was observed between the nesfatin, CRP, and WBC levels and the TBSA at the time of hospitalization of the patients (respectively r = 0.242, P = 0.190; r = 0.117, P = 0.531; r = 0.138, P = 0.46). The most predominant bacterial isolate was Pseudomonas aeruginosa 16 (51.6%) followed by Methicillin resistant Staphylococcus aureus (MRSA) 7 (22.6%), Escherichia coli 5 (16.2%), Methicillin sensitive Staphylococcus aureus (MSSA) 3 (9.7%).

**Table 1.** Demographic and Clinical Characteristics of the Burn Patients and Controls Includedin the Study

Patients, (n = 31), No. (%)	
Male	15(48.4)
Female	16 (51.6)
**Controls, (n = 20), No.**	
Male	7
Female	13
**Age, y, Mean ± SD **	
Patients	15.0 ± 18.6
Controls	15.6 ± 18.8
**Extend of injury (%TBSA ^a^ burned), (range 11-42), Mean ± SD**	23.4 ± 9.2
**The mean length of hospital stay, day, (range 7-100), Mean ± SD**	30.2 ± 19.8

^a^Abbreviation: TBSA, total body surface area

**Table 2.** Nesfatin, CRP, and WBC levels Measured During the Hospitalization of the Patients with Burn Infection and the Values of the Control Group

Factors	Patients (admission of hospital)	Control	P value
**Nesfatin, ng/ml, Mean ± SD**	2.344 ± 0.837	7.252 ± 2.129	0.000
**CRP ** **^a^** **, Mean ± SD**	87.4 ± 75.1	4.0 ± 1.3	0.000
**WBC ** **^a^** **, Mean ± SD**	18126 ± 7643	7230 ± 1450	0.000

^a^Abbreviations: CRP, C-reactive protein; WBC, white blood cell

**Table 3.** The WBC, CRP, and Nesfatin Levels of Patients at the Time of Hospitalization and At Discharge from the Hospital

Factors	Admission of hospital	Discharge from hospital	P value
**Nesfatin, ng/ml, Mean ± SD**	2.344 ± 0.837	4.711 ± 2.268	0.000
**CRP ** **^a^** **, Mean ± SD**	87.4 ± 75.1	27.7 ± 71.1	0.000
**WBC ** **^a^** **, Mean ± SD**	18126 ± 7643	9523 ± 4092	0.000

^a^Abbreviations: CRP, C-reactive protein; WBC, white blood cell

## 5. Discussion

Burn injury causes both metabolic and physiological disruptions (9). Burn patients are subjected to the stress of large open wounds and dressing changes, but they are also prone to metabolic derangements, sleep deprivation, and infections throughout their hospitalization (9, 10). Since burns cause damage to the skin integrity, microorganisms can easily settle on the damaged skin (9, 11). The systemic inflammatory response after a severe burn injury leads to hypermetabolism and thus to protein degradation and catabolism. The hypermetabolic state is characterized by futile protein utilization that results in induction of a dynamic hypercatabolic state concurrent with altered cytokine expression (12, 13). Adding infection to this increased catabolism in patients with burns necessitates an increase in the required diet and calories. However, diminishing appetite is known to occur in patients with burn infection (1). The mediators of this maladaptive response are not clearly understood. Nesfatin-1 is a protein that regulates feeding behavior and is compatible with the concept of the brain-adipose axis (6, 14). The exact mechanism that links diminishing appetite and nesfatin-1 levels in patients with burn infection is not known. Cytokines can stimulate nesfatin-1 release and because inflammation is a common complication in burn injuries patients, nesfatin-1 stimulated by inflammation due to burn wounds could conceivably interfere with food intake control and energy homeostasis. However, burns and especially those associated with infections are known to induce systemic inflammatory responses. (14-17). The data presented here suggest that serum nesfatin-1 levels do not increase in response to inflammation. The present report is the first to describe nesfatin-1 serum concentrations after burn injuries. Several new findings from this pilot study have demonstrated that: First, significantly lower serum nesfatin-1 levels were found in patients with burn infection than in the healthy control group. Second, nesfatin-1 levels significantly increased with the treatment of burn and infection, and approached normal control levels. Third, no correlation could be found between the serum nesfatin level and WBC and CRP levels with acute phase reactants. Fourth, no correlation was found between the serum nesfatin-1 levels and TBSA. This result was not the one expected while planning the study. Since no previous studies have been performed in this connection, we were unable to compare our results directly with other study results. However, Saldanha et al. reported unpublished results of serum nesfatin-1 level in patients with chronic kidney disease that were as low as those in patients with burn infection (16) Saldanha et al. were unable to explain this result, but like us, they also thought that serum nesfatin-1 level would have been increased in response to inflammation. We are also unable to explain why the serum nesfatin-1 level was significantly lower in the patients with burn than in the control group in our study. Yoshida et al. showed that although stress activated the central nesfatin-1 system, the peripheric nesfatin-1 level was not activated by stress (17). Therefore, we considered that this could be related to the expression of nesfatin-1 in the CNS and the periphery. We think that the central nesfatin-1 system should be taken into consideration, rather than the peripheral nesfatin-1 system, when considering the regulation of appetite in a with burns and particularly those accompanied by infection. The Other explanation of the observed negative correlation between nesfatin-1 and burn wound infection suggests that nesfatin-1 may indicate the possible contribution of nesfatin-1 to the energy homeostasis. An appropriate research direction would be to focus on in vivo and vitro studies in this area and on treatment targets related to appetite and nutrition in patients with burn infection.

## References

[1] Hobson KG, Havel PJ, McMurtry AL, Lawless MB, Palmieri TL, Greenhalgh DD (2004). Circulating leptin and cortisol after burn injury: loss of diurnal pattern.. J Burn Care Rehabil..

[2] van de Goot F, Krijnen PA, Begieneman MP, Ulrich MM, Middelkoop E, Niessen HW (2009). Acute inflammation is persistent locally in burn wounds: a pivotal role for complement and C-reactive protein.. J Burn Care Res..

[3] Albayrak Y, Cakir C, Albayrak A, Aylu B (2011). A comparison of the morbidity and mortality of tandir burns and non-tandir burns: experience in two centers.. Ulus Travma Acil Cerrahi Derg..

[4] Rameshwar L.B., Raj KG, Suhas CS, Eiman M, Mohammed KE (1998). Burn septicaemia: an analysis of 79 patients.. Burns..

[5] Ogiso K, Asakawa A, Amitani H, Nakahara T, Ushikai M, Haruta I (2011). Plasma nesfatin-1 concentrations in restricting-type anorexia nervosa.. Peptides..

[6] Oh IS, Shimizu H, Satoh T, Okada S, Adachi S, Inoue K (2006). Identification of nesfatin-1 as a satiety molecule in the hypothalamus.. Nature..

[7] Stengel A, Tache Y (2010). Nesfatin-1--role as possible new potent regulator of food intake.. Regul Pept..

[8] Aydin S, Dag E, Ozkan Y, Arslan O, Koc G, Bek S (2011). Time-dependent changes in the serum levels of prolactin, nesfatin-1 and ghrelin as a marker of epileptic attacks young male patients.. Peptides..

[9] Palmieri TL, Levine S, Schonfeld-Warden N, O'Mara MS, Greenhalgh DG (2006). Hypothalamic-pituitary-adrenal axis response to sustained stress after major burn injury in children.. J Burn Care Res..

[10] Aller MA, Arias JI, Alonso-Poza A, Arias J (2010). A review of metabolic staging in severely injured patients.. Scand J Trauma Resusc Emerg Med..

[11] Yavuz A, Ayse A, Abdullah Y, Belkiz A (2011). Clinical and demographic features of pediatric burns in the eastern provinces of Turkey.. Scand J Trauma Resusc Emerg Med..

[12] Albayrak A, Uyanik MH, Odabasoglu F, Halici Z, Uyanik A, Bayir Y (2011). The effects of diabetes and/or polymicrobial sepsis on the status of antioxidant enzymes and pro-inflammatory cytokines on heart, liver, and lung of ovariectomized rats.. J Surg Res..

[13] Finnerty CC, Jeschke MG, Herndon DN, Gamelli R, Gibran N, Klein M (2008). Temporal cytokine profiles in severely burned patients: a comparison of adults and children.. Mol Med..

[14] Shimizu H, Ohsaki A, Oh IS, Okada S, Mori M (2009). A new anorexigenic protein, nesfatin-1.. Peptides..

[15] Merali Z, Cayer C, Kent P, Anisman H (2008). Nesfatin-1 increases anxiety- and fear-related behaviors in the rat.. Psychopharmacology (Berl)..

[16] Saldanha JF, Carrero JJ, Mafra D (2011). The possible role of nesfatin-1 on appetite regulation in hemodialysis patients.. Med Hypotheses..

[17] Yoshida N, Maejima Y, Sedbazar U, Ando A, Kurita H, Damdindorj B (2010). Stressor-responsive central nesfatin-1 activates corticotropin-releasing hormone, noradrenaline and serotonin neurons and evokes hypothalamic-pituitary-adrenal axis.. Aging (Albany NY)..

